# Mushroom intoxication, a fatal condition in Romanian children: Two case reports

**DOI:** 10.1097/MD.0000000000017574

**Published:** 2019-10-11

**Authors:** Cristina Oana Mărginean, Lorena Elena Meliţ, Maria Oana Mărginean

**Affiliations:** a Department of Paediatrics; b Department of Paediatric Cardiology, University of Medicine, Pharmacy, Sciences and Technology, Târgu Mures, Romania.

**Keywords:** acute liver failure, children, mushroom intoxication

## Abstract

**Rationale:**

Approximately 5000 species of wild mushroom are reported worldwide, of which 100 are documented as poisonous and <10 are fatal. The clinical picture of patients with wild mushroom intoxication depends mostly on the type of ingested mushroom, ranging from mild gastrointestinal symptoms to organ failure and death.

**Patient concerns:**

We report 2 children, sister and brother admitted in our clinic for gastrointestinal symptoms: abdominal pain, nausea, vomiting, and diarrhea after wild mushroom ingestion.

**Diagnosis:**

The laboratory tests revealed hepatic cytolysis syndrome, hyperbilirubinemia, impaired coagulation status, hypoalbuminemia, hypoglycemia, and electrolytic unbalances in both cases. Abdominal ultrasound showed hepatomegaly and ascites.

**Intervention:**

After admission, both cases received penicillin by vein, activated charcoal, liver protectors, glucose, and electrolytes perfusions. Nevertheless, their status worsened and required the transfer to the pediatric intensive care unit for appropriate supportive measure. Therefore, therapeutic plasma exchange was initiated along with N-acetyl cysteine and hemostatic drugs.

**Outcomes:**

Despite all these therapeutic interventions, both cases developed hepatorenal syndrome and died after a couple of days from ingestion.

**Lessons:**

Mushroom poisoning remains a public health problem in developing countries. Preventable strategies and education regarding the consumption of wild type mushrooms are essential for decreasing the morbidity and mortality rates in these areas.

**Key Points**

- Mushroom poisoning remains a public health problem in developing countries. Preventable strategies and education regarding the consumption of wild type mushrooms are essential for decreasing the morbidity and mortality rates in these areas.

## 1 Introduction

A wide variety of wild mushrooms grow spontaneously in forests and meadows especially after rainfalls, and they are frequently consumed by the local population with a potential fatal outcome. The current back-to-nature and organic food movements have led to an increase in wild mushroom consumption.^[1]^ There were reported approximately 5000 species of mushrooms worldwide, but only 20% to 25% have been named, of which 3% were identified as poisonous.^[2]^ The most toxic native species are *Amanita phalloides*, also known as death cap; *A virosa* or destroying angel; and *Cortinarius speciosissimus* or web caps.^[3]^ Thus, amatoxin-containing species that cause fulminant hepatic cytolysis, and *Cortinarius* species leading to acute renal failure, are the most frequent leading causes of death due to mushrooms intoxication.

The diagnosis of mushroom intoxication must be based on anamnesis, the identification of wild mushroom type if possible, and laboratory tests. In these cases, the anamnesis depends mostly on the physician's communications skills since the patient is often under the hallucinogenic effect of these mushrooms.^[4]^ The clinical picture of patients with wild mushroom intoxication depends mostly on the type of ingested mushroom, ranging from mild gastrointestinal symptoms, such as abdominal pain, nausea, vomiting, and diarrhea; to organ failure and death due to major cytotoxic effects that can occur in rare cases.^[1]^ Other factors that influence the toxicity are the season, the geographic location, the age of the mushroom, and the preparation pattern before ingestion.^[1]^ The identification of the wild mushroom is very difficult in most of the cases, and thus the patient's symptoms must orientate the treatment in all cases.^[5]^ Based on the reported mushroom intoxication studies, 4 groups of individuals were described: wild-mushroom foragers, accidental ingestion in young children, individuals aiming a hallucinatory effect, and those attempting suicide.^[1]^ Therefore, consumption of poisonous mushrooms may lead to allergic gastroenteritis, psychological effects, and acute liver injure, often fatal.^[5–7]^ Nevertheless, hepatic cytolysis due to mushroom poisoning must be differentiated by other causes of poisoning^[8]^ or other conditions that involve liver impairment.^[9–16]^ Acute liver failure due to mushroom poisoning is a result of the toxic effect of the hepatotoxin found in wild types of mushrooms. Most often, this hepatotoxin is an amatoxin resulting in severe hepatic cytolysis, being responsible in 90% of the cases for the fatal outcome of these patients.^[17]^ Thus, every year up to 100 fatalities due to mushroom poisoning are reported worldwide, and most of them are caused by acute liver failure.^[18]^

Multiple therapeutic approaches have been used in severe cases of wild mushroom poisoning, such as plasmapheresis; the molecular adsorbent recirculating system (MARS) and different antidotes reported as useful, such as silibin, penicillin G, N-acetylcysteine, or high doses of ascorbic acid.^[17]^ Despite all these therapeutic attempts, the occurrence of acute liver and renal failure leads eventually to a fatal outcome in these patients, most-likely due to *Amanita* poisoning.

We report 2 cases of fatal mushroom intoxication in 2 Romanian children, a 9-year-old girl and her 5-year-old brother, to underline the potentially fatal outcome of this condition in these ages.

The informed written consent was obtained from the patients’ mother before publication of these cases.

## 2 Case reports

### 2.1 Case 1

#### 2.1.1 Presenting concerns

We report the case of 9-year-old girl, with a poor socioeconomic level, from a rural area, admitted in our clinic for abdominal pain, nausea, vomiting, and diarrheic stools after wild mushroom ingestion approximately 24 hours before the admission. The mushrooms were gathered from the forest by an older relative, and improperly fried by the patient herself. Her family and personal history did not reveal any significant data.

#### 2.1.2 Clinical findings

The clinical examination at the time of admission pointed out altered general status, dry lips, and diminished cutaneous turgor, abdominal tenderness, repeated vomiting, and accelerated bowel movements, weight: 30 kg.

#### 2.1.3 Diagnosis focus and assessment

The laboratory tests at the time of admission revealed mildly elevated liver enzymes: aspartate aminotransferase (AST) 120 U/L, alanine aminotransferase (ALT) 94 U/L, and hypoglycemia [blood glucose (Glu) 53 mg/dL]. The levels of bilirubin and coagulations tests were within normal ranges. Her symptoms worsened within the next 24 hours after admission and her general status altered considerably. The laboratory tests repeated after approximately 36 hours from admission showed: severe hepatic cytolysis syndrome (AST 8543 U/L, ALT 7624 U/L), elevated levels of total bilirubin (TBi 4.924 mg/dL, direct bilirubin [DBi] 4.405 mg/dL), and creatinfosfokinase 2 (1431 U/L), hypoalbuminemia (Alb 2.84 g/dL), hypoglycemia (Glu 22 mg/dL), hyponatremia [sodium (Na) 111 mmol/L], impaired renal function (creatinine 1.04 mg/dL), and altered coagulation tests [activated partial thromboplastin time (APTT) 140.6 seconds, international normalized ratio—INR unmeasurable]. The abdominal ultrasound revealed hepatomegaly and ascites. The patient presented a tonic clonic seizure in context of hypoglycemia and she was transferred to the pediatric intensive care unit (PICU).

#### 2.1.4 Therapeutic focus and outcome

After admission, we initiated treatment with penicillin G by vein; glucose, electrolytes, and amino acids perfusion; and activated charcoal. Nevertheless, the patient's general status worsened requiring the transfer to the PICU, where the previously mentioned therapy was continued, but therapeutic plasma exchange was also initiated along with N-acetylcysteine hemostatic drugs, such as coagulation factor VII and vitamin K. Moreover, she developed digestive hemorrhage requiring multiple blood transfusions. We took into account liver transplantation, but the patient's condition did not allow this therapeutic option. Despite all our efforts, the death occurred after approximately 60 hours from admission.

### 2.2 Case 2

#### 2.2.1 Presenting concerns

The second case describes the girl's little brother, a 5-year-old boy admitted in our clinic for the same symptoms as his sister due to wild mushroom ingestion, but approximately 24 hours later. His family and personal history were also negative.

#### 2.2.2 Clinical findings

The clinical examination at the time of admission revealed influenced general status, pallor, dry lips, and diminished cutaneous turgor, abdominal tenderness, and diarrheic stools, weight 16.5 kg.

#### 2.2.3 Diagnostic focus and assessment

The laboratory tests at the time of admission were worse in comparison the his sister, revealing more elevated levels of liver enzymes (AST 597 U/L, ALT 784 U/L), hyperbilirubinemia (TBi 4.192 mg/dL, DBi 3.747 mg/dL), hyponatremia (Na 133 mmol/L), hypopotassemia (K 3.2 mmol/L), hypoalbuminemia (Alb 2.55 mg/dL), and altered coagulation tests (APTT 73.1 seconds, INR 3.68). The abdominal ultrasound showed hepatomegaly and ascites. After approximately 24 hours from admission, the hepatic cytolysis syndrome worsened (AST 1960 U/L, ALT 2544 U/L), and he was transferred to the PICU.

#### 2.2.4 Therapeutic focus and outcome

Similar to the case presented above, the little brother also received penicillin G by vein, glucose, electrolytes, and amino acids perfusion, and activated charcoal within the first hour after admission. Nevertheless, his condition continued to worsen and therapeutic plasma exchange along with the same supportive measures as in the case of his sister, were initiated in the PICU. Despite these therapeutic efforts, his liver enzymes continued to increase (AST 4386.7 U/L, ALT 5156.2 U/L) associating also renal dysfunction (creatinine 2.18 mg/dL). Liver transplantation was not a viable option under these circumstances. Unfortunately, the patients died after approximately 100 hours from the time of admission.

## 3 Discussions

The collection and consumption of wild mushroom remains a lifelong tradition in Romania as in other European countries. Moreover, this habit is the most common among people with a poor socioeconomic level, representing a major health risk in those living in rural areas.^[1]^ Thus, mushroom poisoning might be considered a real public health problem especially in developing countries. Similar to other severe conditions that benefit from important screening programs,^[19,20]^ effective preventable strategies and educational programs should be elaborated and implemented in these areas to decrease the morbidity and mortality related to this condition. Depending on geographic area, mushroom poisoning occurs in different times of the year since their development is strongly related to the increased degree of humidity and relatively high temperature. Thus, this condition might occur from June to December.^[21]^ A recent study from Turkey pointed out that >50% of the patients diagnosed with mushroom poisoning consumed them in the early summertime.^[1]^ The cases described above originated from a rural area and had a poor socioeconomic status. Similar to the aforementioned study, the consumption in our cases occurred in July.

The majority of the ingested mushrooms present no toxic effect or only mild gastrointestinal ones.^[22]^ Depending on the time of clinical symptoms onset, mushroom toxicity can be divided into early, which appears within the first 6 hours after ingestion, and delayed, occurring between 6 hours and 20 days after ingestion.^[5]^ In both our patients, the symptoms occurred after >20 hours from ingestion, being therefore classified as delayed toxicity. The latency period of symptoms after ingestion is essential for the patient's prognosis. Thus, late-toxicities usually result in liver and renal failure, being related to amatoxin poisoning.^[1]^ It is well documented that the onset of symptoms in case of mushroom poisoning with amatoxins occurs at 6 to 24 hours after ingestion.^[23]^ It is also important to mention that in case of *Amanita* poisonings, impaired coagulation status, encephalopathy, and hepatorenal syndrome occur along with liver failure.^[24]^ Both brothers described above presented impaired coagulation status and renal dysfunction before death. Fortunately, delayed toxicity usually happens in <90% of the patients with this condition.^[1]^ In case of children, poisoning is a frequent preventable cause of morbidity and mortality since most of the cases happen accidentally. Thus, a study performed on 122 children with acute poisoning identified mushroom ingestion in 8.2% of the cases.^[25]^ Unfortunately, in our cases the consumption was not accidental. Moreover, it seems that children are more susceptible than adults to mushroom toxicity, most likely due to metabolic differences and immature liver function.^[26]^ The early diagnosis and treatment are mandatory for increasing the survival rates in patients with mushroom poisoning.

The clinical features in case of *Amanita* poisoning are characterized by 4 stages. Thus, the first stage is defined by a latent period (6–40 hours) with no apparent symptoms, whereas the second stage is characterized by gastrointestinal symptoms, such as abdominal pain, nausea, vomiting, or loose stools.^[17]^ In the third stage, the patient experiences an apparent improvement of clinical symptoms contrasting with the severe deterioration of laboratory parameters.^[17]^ The last stage of this intoxication is characterized by the association between acute liver failure, hepatic encephalopathy, hyperbilirubinemia, impairment of coagulation status, acute renal failure, and hypoglycemia.^[17]^ Both our cases expressed this pattern of mushroom intoxication. The impairment of coagulations test may result in different types of hemorrhage. In the first case presented above, digestive hemorrhage burdened the patient's evolution and management. Nevertheless, digestive hemorrhage might appear also in case of other gastrointestinal disorders along with abdominal pain, nausea, or vomiting.^[27,28]^

The difficulties related to the management of patients with mushroom poisoning depend mostly on the type of ingested mushroom and the patient's symptoms. Since amatoxin has no specific antidote, multiple therapeutic options are reported as useful in these patients: benzylpenicillin, silymarin complex, thioctic acid, or antioxidant drugs (cimetidine, ascorbic acid, and N-acetyl cysteine). In more severe cases, therapeutic plasma exchange, extracorporeal albumin dialysis, and MARS might represent a bridge until liver transplantation. Unfortunately, both our patients died despite all therapeutic efforts. Based on our patients’ symptoms, evolution and fatal outcome, most-likely *A phalloides* was the ingested type of mushroom.

## 4 Conclusions

Even though fatal cases due to wild mushroom poisoning are rare, this condition must be considered life threatening in all cases. Thus, early diagnosis and treatment are essential for the outcome of these patients. Educational and preventive strategies are mandatory especially in developing countries to decrease morbidity and mortality related to wild mushroom ingestion.

## Acknowledgments

The authors express their sincere gratitude to Dr Pitea Ana Maria and Pediatric Intensive Care Unit for the involvement in the management of these 2 cases.

## Author contributions

**Conceptualization:** Lorena Elena Melit.

**Data curation:** Maria Oana Mărginean.

**Formal analysis:** Cristina Oana Mărginean.

**Investigation:** Cristina Oana Mărginean, Lorena Elena Melit.

**Methodology:** Cristina Oana Mărginean, Lorena Elena Melit.

**Resources:** Cristina Oana Mărginean.

**Supervision:** Cristina Oana Mărginean, Maria Oana Mărginean.

**Validation:** Cristina Oana Mărginean, Maria Oana Mărginean.

**Writing – original draft:** Cristina Oana Mărginean, Lorena Elena Melit, Maria Oana Mărginean.

**Writing – review and editing:** Cristina Oana Mărginean, Lorena Elena Melit, Maria Oana Mărginean.
